# Optimized Doxycycline-Inducible Gene Expression System for Genetic Programming of Tumor-Targeting Bacteria

**DOI:** 10.1007/s11307-021-01624-x

**Published:** 2021-08-17

**Authors:** Dinh-Huy Nguyen, Sung-Hwan You, An-Trang Ngoc Vo, Hien Thi-Thu Ngo, Khuynh Van Nguyen, Mai Thi-Quynh Duong, Hyon E. Choy, Miryoung Song, Yeongjin Hong, Jung-Joon Min

**Affiliations:** 1 Institute for Molecular Imaging and Theranostics, Chonnam National University Medical School, Gwangju, 61469 Republic of Korea; 2 Department of Molecular Medicine (BrainKorea21 Plus), Chonnam National University Graduate School, Gwangju, 61469 Republic of Korea; 3 Department of Microbiology, Chonnam National University Medical School, Gwangju, 61469 Republic of Korea; 4 Department of Bioscience and Biotechnology, Hankuk University of Foreign Studies, Yongin, South Korea; 5 Department of Nuclear Medicine, Chonnam National University Medical School, Jeonnam Gwangju, 61469 Republic of Korea

**Keywords:** Tet system, *Salmonella*, Theranostics, Bacteria-mediated gene therapy

## Abstract

**Purpose:**

In the programming of tumor-targeting bacteria, various therapeutic or reporter genes are expressed by different gene-triggering strategies. Previously, we engineered pJL87 plasmid with an inducible bacterial drug delivery system that simultaneously co-expressed two genes for therapy and imaging by a bidirectional *tet* promoter system only in response to the administration of exogenous doxycycline (Doxy). In this multi-cassette expression approach, *tetA* promoter (P_*tetA*_) was 100-fold higher in expression strength than *tetR* promoter (P_*tetR*_). In the present study, we developed pJH18 plasmid with novel Doxy-inducible gene expression system based on a *tet* promoter.

**Procedures:**

In this system, Tet repressor (TetR) expressed by a weak constitutive promoter binds to *tetO* operator, resulting in the tight repression of gene expressions by P_*tetA*_ and P_*tetR*_, and Doxy releases TetR from *tetO* to de-repress P_*tetA*_ and P_*tetR*_.

**Results:**

In *Salmonella* transformed with pJH18, the expression balance of bidirectional *tet* promoters in pJH18 was remarkably improved (P_*tetA*_:P_*tetR*_ = 4~6:1) compared with that of pJL87 (P_*tetA*_:P_*tetR*_ = 100:1) in the presence of Doxy. Also, the expression level by novel *tet* system was much higher in *Salmonella* transformed with pJH18 than in those with pJL87 (80-fold in *rluc8* and 5-fold in *clyA*). Interestingly, pJH18 of the transformed *Salmonella* was much more stably maintained than pJL87 in antibiotic-free tumor-bearing mice (about 41-fold), because only pJH18 carries *bom* sequence with an essential role in preventing the plasmid-free population of programmed *Salmonella* from undergoing cell division.

**Conclusions:**

Overall, doxycycline-induced co-expression of two proteins at similar expression levels, we exploited bioluminescence reporter proteins with preclinical but no clinical utility. Future validation with clinically compatible reporter systems, for example, suitable for radionuclide imaging, is necessary to develop this system further towards potential clinical application.

**Supplementary Information:**

The online version contains supplementary material available at 10.1007/s11307-021-01624-x.

## Introduction

The disordered tumor microenvironment is responsible for oxygen deficiency, excessive nutrient leakage, and immune privilege, which can provide a niche in which bacteria such as *Clostridium* [1], *Bifidobacterium* [2, 3], *Listeria* [4, 5], *Escherichia coli* [6, 7], and *Salmonella* [8, 9] can survive. Bacteria are well-suited for distinguishing tumor tissue from normal organs because they specifically colonize tumors and proliferate within them [10, 11]. We previously developed an attenuated strain of *Salmonella typhimurium* that was defective in guanosine 5′-diphosphate-3′-diphosphate synthesis (∆ppGpp *Salmonella*, SAM) and which showed preferential accumulation in tumors, resulting in bacterial numbers that were more than 10,000-fold higher in tumor tissue than in healthy organs [12].

As attenuated bacteria alone are often unable to eradicate malignant tumors, various bacterial species have been subjected to genetic programming to develop tumor-selective protein–drug factories [8, 9, 12–15]. Various effector systems have been explored for their ability to express and deliver therapeutic payloads to cancer [11]. Because bacteria tend to localize initially, but transiently, in the liver and spleen [11, 16], various inducible effector systems that utilize external gene triggers such as l-arabinose [8, 9, 12, 17], salicylate [18], γ-irradiation [19], and tetracycline [8] have been actively developed to maximize intratumoral effects while minimizing systemic toxicity.

Tetracycline and its analogues such as doxycycline (Doxy) exhibit several properties of an ideal inducer: they regulate gene expression at very low concentrations (nmol/l range) and are therefore nontoxic at the necessary levels; they show good bioavailability as they can penetrate both bacterial and animal cells; their stability is suitable for the time courses required for a therapeutic effect; and they are well tolerated in humans, being widely used as antibiotics [8]. The bidirectional *tet* expression system is simultaneously regulated by the P_*tetA*_ and P_*tetR*_ promoters, which are induced by tetracycline or Doxy [8, 20, 21]. We previously reported a Doxy-inducible bacterial drug delivery system (pJL39) [8] in which a reporter gene and a therapeutic gene under the influence of bidirectional *tet* promoters were inserted to visualize the targeting process and deliver therapeutic drugs. This inducible system co-expressed dual genes only in response to the administration of Doxy, which was regulated in a dose- and time-dependent manner. Although this inducible system revealed advantages for facilitating controllable therapeutic gene expression in small animal models, the imbalance in promoter strength between P_*tetA*_ and P_*tetR*_ (P_*tetA*_ is 100-fold stronger than P_*tetR*_) is a limiting factor that may hamper achievement of maximal transgene expression levels from the bacteria. The open reading frames (ORFs) of the target gene under P_*tetR*_ are located distal to *tetR* gene, and distal ORFs are often expressed less efficiently than proximal ones; P_*tetR*_ is often relatively less expressed, although there can also be considerable variation according to the bacterial strain and growth conditions.

In the absence of selection pressure, bacteria typically fail to maintain the expression plasmid, particularly in infected animals [22, 23]. Therefore, we employed the *bom* sequence to ensure stable plasmid maintenance during bacterial growth in an infected mouse model, without the requirement for antibiotic pressure. *Bom*, a functional sequence for *cis* mobility, contains *nic* (known as the origin of transfer, *oriT*), a site at which a site-specific nick is formed by the relaxation of complex proteins to initiate plasmid mobilization [24, 25]. In fact, depletion of the *bom* or *ori*T sequence resulted in a 95–99 % reduction in the transfer frequency of plasmids in a study of DNA plasmid transfer origin [24].

In this study, we modified the previously reported system pJL39 to promote transcriptional activity of target genes under P_*tetR*_ [8] and developed a novel Doxy-inducible system (pJH18). In the present system, the *tetR* gene was decoupled from P_*tetR*_ and placed under the control of a weak constitutive promoter (P_*araB*_), resulting in direct control of target genes by P_*tetR*_, rather than transcriptional read-through from the *tetR* transcript, as in pJL39 [8]. Furthermore, the location of *bom* sequence, which is required for plasmid mobilization [25, 26], plays a critical role in preventing bacterial plasmid loss under normal conditions, resulting in the maintenance of durable protein expression. The present Doxy-inducible system enabled strict regulation and enhanced expression of transgenes in response to a low dose of exogenous Doxy, which achieved the desired biological effect in small animal models.

## Results

### Engineering of the Novel Doxy-Inducible Bidirectional Expression System

**Fig. 1.**
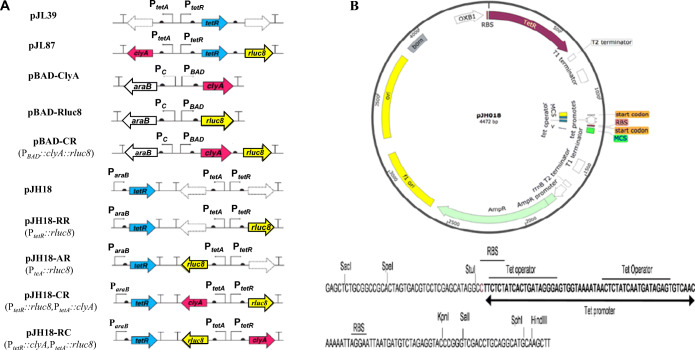
Schematic diagram of the plasmids used in this study. **A** Simple description of the plasmids used in this study. Solid big arrows, open reading frames; curved arrows, promoters; T marks, terminators; semicircles, regulator binding sites. **B** Map and multiple cloning sites (lower part) of the pJH18 vector. A detailed description is provided in the Materials and Methods. MCS, multiple cloning site; RBS, ribosomal binding site; bp, base pairs.

To improve the expression balance between P_*tetA*_ and P_*tetR*_ and the transfer frequency of the plasmids (Fig. 1), we modified pJL39 to generate pJH18 in which TetR was controlled by the weak constitutive promoter P_*araB*_, and bidirectional *tet* promoters, P_*tetA*_ and P_*tetR*_, could be used for simultaneous expression of two cargo genes (Fig. 1A). Furthermore, *bom* sequence was also cloned in this plasmid for its ability to facilitate bacterial maintenance in antibiotic-free conditions (Fig. 1B). Using pJH18, we constructed four plasmids in which a reporter gene (*rluc8* for *Renilla* luciferase variant 8) and/or a therapeutic gene (*clyA* for cytolysin A) were placed under the control of P_*tetA*_ or P_*tetR*_; pJH18-RR encoding *rluc8* under *P*_*tetR*_, pJH18-AR encoding *rluc8* under P_*tetA*_, pJH18-CR encoding *clyA* and *rluc8* under P_*tetA*_ and P_*tetR*_, respectively, and pJH18-RC encoding *rluc8* and *clyA* under P_*tetA*_ and P_*tetR*_, respectively (Fig. 1A).

**Fig. 2.**
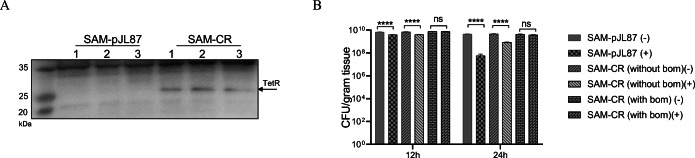
Measurement of TetR expression and *bom* gene activity in pJH18 transformants. **A** Measurement of TetR expression. ΔppGpp *Salmonella* were transformed with pJH18-CR (SAM-CR) or pJLS7 (SAM-pJL87). The transformed bacteria were freshly cultured to exponential (OD600 ~0.3 and 0.6) and stationary (overnight culture) phases in ampicillin-containing LB broth. Bacterial pellets (8 × 10* CFU/ml) were dissolved in sample buffer, separated in SDS-PAGE and stained with Coomassie blue (upper panel). TetR proteins (~25 kDa) were detected with western blot using their antibodies (bottom panel). 1, OD600 0.3; 2, OD600 0.6; 3, overnight culture. **B** Measurement of *bom* sequence activity. Bacteria were cultured overnight in the absence of ampicillin, and 10^−6^ diluted bacteria were spread on agar plates supplemented with ampicillin and cultured for 1 day. Colonies on each plate were counted, and the total bacterial numbers are depicted after compensation for the dilution factor. +, with ampicillin; -, without ampicillin (*n* = 3 per group. ****P < 0.0001; ns, not significant, two-way analysis of variance (ANOVA) with Bonferroni’s multiple comparison test). All data are shown as mean S.E.M.

We transformed ∆ppGpp *Salmonella* with the pJH18-CR (SAM-CR) or pJL87 (SAM-pJL87) plasmid encoding *clyA* and *rluc8* under P_*tetA*_ and P_*tetR*_, respectively [8]. TetR expression and *bom* activity were compared between the two transformed bacteria. In SAM-CR, TetR proteins (23 kDa) were constitutively expressed in every growth time of Doxy-free condition (Fig. 2A), whereas in SAM-pJL87, TetR was undetectable at any growth time point. We note that TetR expression relied on Doxy in pJL87, as it is expressed by P_*tetR*_ [8]. To assess plasmid maintenance in SAM-pJL87 (without *bom*) and SAM-CR (with or without *bom*), these transformants were spread onto agar plates after overnight culture in the absence of ampicillin. After 24 h of culture, SAM-CR (with *bom*) showed no significant change of colony numbers in the presence or absence of ampicillin, whereas only 12.9 % of the SAM-CR (without *bom*) and 1.7 % of the SAM-pJL87 colonies remained in the presence of ampicillin compared with those in the absence (Fig. 2B). This result indicates that pJH18 is stably maintained for 24 h, even without antibiotics because it contains *bom* sequence.

**Fig. 3.**
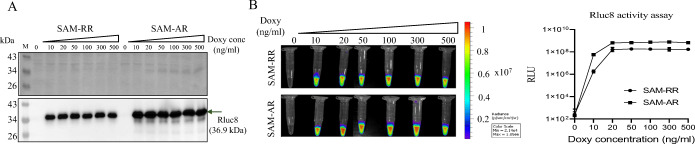
Comparison of Rluc8 expression by P_*tetA*_ and P_*tetR*_. SAM were transformed with pJH18-RR (SAM-RR) or pJH18-AR (SAM-AR). After culture to OD600 0.5~0.7, the bacteria were added with the indicated concentrations of Doxy and further cultured for 4 h. The same numbers of bacteria (8 × 10^8^ CFU/ml) were harvested by centrifugation and used for further analysis. **A** Western blot analysis of Rluc8. After dissolving in sample buffer, the proteins in the bacterial pellets were separated on two SDS-PAGE gels. One gel was stained with Coomassie blue dye (upper panel), whereas the proteins in the other gel were transferred to a nitrocellulose membrane and blotted with anti-Rluc8 antibody. M, marker. **B** Measurement of luciferase activity. Bacterial pellets were dissolved in PBS with coelenterazine (1 μg/ml). Photographs (right) and light emission rates (left) of each sample were acquired by cooled CCD camera.

Next, we evaluated Doxy-inducible expression of a reporter gene in cultured SAM transformed with pJH18-RR (SAM-RR) or pJH18-AR (SAM-AR). Doxy-induced Rluc8 (36.9 kDa) expression was measured in bacterial cultures after 4-h induction with various Doxy concentrations (Fig. 3). The luciferase activity by Rluc8 was specifically identified only in the presence of Doxy. Doxy-induced Rluc8 expression could be detected as a saturated amount in western blot analysis even at the lowest Doxy concentration (10 ng/ml) (Fig. 3A). The Rluc8 protein level was 2- to 6-fold higher under P_*tetA*_ than under P_*tetR*_. The western blot result was consistent with that of a luciferase activity assay (Fig. 3B), with luciferase activity being detected even with 10 ng/ml of Doxy and being saturated at 20 ng/ml of Doxy. This luciferase activity was 3-fold higher with P_*tetA*_ than with P_*tetR*_ (Fig. 3B).

### Enhanced Protein Expression Induced by Doxy in SAM Transformed with pJH18

**Fig. 4.**
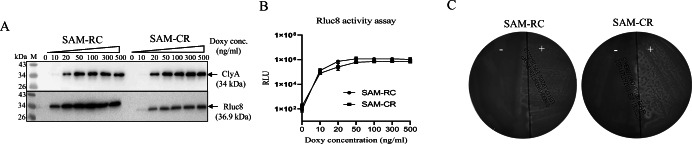
Expression of ClyA and Rluc8 in pJH18 plasmid. SAM were transformed with pJH18-CR or pJH18-RC. After culture to OD600 0.5~0.7, bacteria were added with the indicated concentration of Doxy and further cultured for 4 h. The same numbers of bacteria (8 × 10^8^ CFU/ml) were harvested with centrifugation and used for further analysis. **A** Western blot analysis of ClyA and Rluc8. After dissolving in sample buffer, the bacterial pellets were separated using two SDS-PAGE gels. The proteins were then transferred to nitrocellulose membranes and blotted with anti-ClyA (upper panel) or anti-Rluc8 (bottom panel) antibodies. **B** Measurement of luciferase activity. Bacterial pellets were dissolved in PBS with coelenterazine (1 μg/ml). Light emission rates of each sample were acquired by cooled CCD camera. **C** Hemolysis assay. After culture with Doxy, bacteria were streaked onto a blood agar plate and cultured overnight. +, with Doxy; -, without Doxy.

Subsequently, we engineered bacteria carrying pJH18-RC (SAM-RC) or pJH18-CR (SAM-CR) and assessed cargo gene expression. In western blot analyses, both Rluc8 (36.9 kDa) and ClyA (34 kDa) proteins could be specifically identified only in the presence of Doxy (Fig. 4A). In SAM-RC and SAM-CR, the expression levels of these proteins were 2- to 6-fold higher under P_*tetA*_ than under P_*tetR*_. This result was consistent with Rluc8 enzyme activity assays (Fig. 4B). The ClyA activity secreted from SAM-RC or SAM-CR was also evaluated using a blood agar plate; SAM-RC and SAM-CR could lyse blood cells only after Doxy induction (Fig. 4C).

**Fig. 5.**
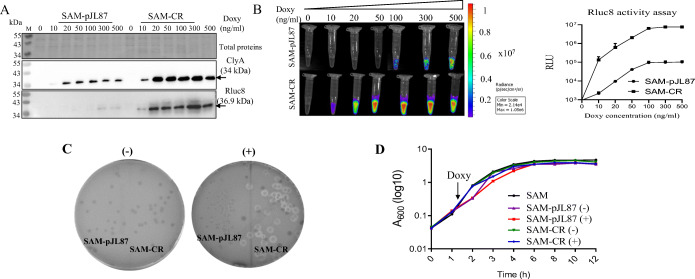
Comparison of cargo expression in SAM-CR and SAM-pJL87. **A** Western blot analysis of ClyA and Rluc8 in SAM-CR and SAM-pJL87. **B** Measurement of luciferase activity in SAM-CR and SAM-pJL87. **C** Hemolysis assay. Bacteria were streaked onto blood agar plates supplemented with Doxy (300 ng/ml) and cultured overnight. +, with Doxy; -, without Doxy. **D** Bacterial growth curve. After culture overnight, 100-fold diluted bacteria were inoculated into fresh media and cultured for 1.5 h, after which Doxy (300 ng/ml) was added and the broth was further cultured. Bacterial growth was measured at OD600 with a spectrophotometer.

We then compared the cargo expression levels of SAM-CR and SAM-pJL87, which express ClyA and Rluc8 under P_*tetA*_ and P_*tetR*_, respectively (Fig. 5). After Doxy induction, the expression of Rluc8 and ClyA was approximately 80-fold and 5-fold stronger in SAM-CR than in SAM-pJL87, respectively (Fig. 5A). In particular, Rluc8 expression under the control of P_*tetR*_ showed a remarkable increase in SAM-CR. The expression difference was also reproduced in Rluc8 activity tests. Rluc8 luciferase activity was 30- to 90-fold higher in SAM-CR than in SAM-pJL87, depending on the concentration of Doxy (Fig. 5B). After Doxy induction, SAM-CR showed stronger hemolytic activity than SAM-pJL87 (Fig. 5C). It should be noted that SAM-CR grew well, similar to non-transformed bacteria, despite the strong expression of ClyA and Rluc8 after Doxy induction (Fig. 5D).

The arabinose-inducible expression system using P_*BAD*_ promoter has been widely used for precise and strong induction of protein expression in bacteria-based cancer therapy [9, 27]. Thus, we assessed cargo expression in SAM transformed with pJH18 or pBAD (Fig. S1). The ClyA and Rluc8 expressions driven by P_*BAD*_ were comparable to those by P_*tetA*_ and P_*tetR*_. The expression of ClyA and Rluc8 proteins in SAM-pBAD-ClyA was 1.4-fold higher than in SAM-RC (P_*tetR*_::*clyA*) but 1.3-fold lower than in SAM-CR (P_*tetA*_::*clyA*) (Fig. S1A). This result was consistent with the Rluc8 activity assay (Fig. S1B). Furthermore, we also found that in the single-cassette-polycistronic pBAD system encoding ClyA and Rluc8 (pBAD-CR (P_*BAD*_*::clyA::rluc8*), Fig. 1A), there was no significant difference in proximal ClyA expression between SAM-pBAD-CR and SAM-CR, but the expression of Rluc8 protein was 3.2-fold lower in SAM-pBAD-CR than that in SAM-CR (Fig. S2A). This result was corresponded with the bioluminescence activity (Fig. S2B). The result indicates that the pJH18 system in the present study is appropriate for balanced expression of two genes.

### *In Vivo* Doxy-Inducible Gene expression in Bacteria of Mouse Tumor Models

**Fig. 6.**
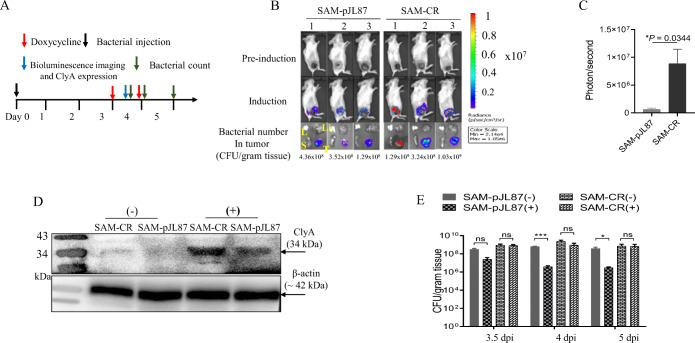
*In vivo* cargo expression by SAM-CR and SAM-pJL87 in tumor xenografts. SAM-CR or SAM-pJL87 (3 × 107 CFU/mouse) were injected *via* the tail vein of BALB/c mice bearing CT26 (three mice per group). At 3 dpi, Doxy (17 mg/kg body weight) was orally administrated. **A** Experimental schedule. **B** Bioluminescence imaging of whole body and *ex vivo* (lung, L; liver, Li; spleen, S; tumor, T). Images were obtained after coelenterazine injection (0.7 mg/kg body weight) *via* tail vein. **C** Measurement of luciferase activity in CT26 xenografts. Light emission amounts were acquired by cooled CCD camera. **D** ClyA identification in tumor tissue by western blot analysis. CT26 tumor tissues were cut out from mice and crushed. The proteins were separated in SDS-PAGE and transferred to nitrocellulose membrane. Western blot was done with anti-ClyA (upper panel) and anti-beta actin (bottom panel) antibodies. +, Doxy administrated mice; -, Doxy non-administrated mice. **E** Measurement of plasmid maintenance in bacteria. After Doxy administrated in bacteria-injected mice, bacteria were isolated from tumor tissues at the indicated time points and re-cultured in agar plates supplemented with or without ampicillin. One day after culture, colony numbers were counted. +, plates with ampicillin; -, plates without ampicillin (*n* = 3 per group, *P* = 0.0123, ***P = 0.0003; ns, not significant, two-way analysis of variance (ANOVA) with Bonferroni’s multiple comparison test). All data are shown as mean + S.E.M.

In our previous study, we demonstrated Rluc8 activity driven by P_*tetR*_ in tumor tissues when SAM-pJL87 was injected into mouse tumor models, but its level was 100-fold lower than that driven by P_*tetA*_ promoter [8]. We evaluated the improvement in the efficiency of gene expression under P_*tetR*_ in SAM-CR by comparing it with that of SAM-pJL87 in CT26 xenograft tumors (Fig. 6). Mice were injected with SAM-CR or SAM-pJL87 *via* the tail vein and were orally administered Doxy (17 mg/kg body weight) at 3 days postinoculum (dpi) (Fig. 6A). Rluc8 activity (driven by P_*tetR*_) in CT26 xenografts treated with SAM-CR was 14-fold higher than in those treated with SAM-pJL87 (Fig. 6 B and C), and ClyA protein expression (driven by P_*tetA*_) was 3-fold higher in tumor tissues treated with SAM-CR than in those treated with SAM-pJL87 (Fig. 6D). These results indicate that the novel Doxy-inducible system pJH18 was able to express dual genes in tumor-targeting bacteria much more efficiently than was pJL87.

Interestingly, there were no significant differences in bacterial numbers in the mouse tumors treated with SAM-CR until 5 dpi, regardless of Doxy induction (Fig. 6E). By contrast, bacterial numbers in mouse tumors treated with SAM-pJL87 decreased more than 10-fold after Doxy induction compared with numbers before Doxy induction at 3.5, 4, and 5 dpi. We speculate that Doxy induction affects plasmid maintenance in the pJL87 system and that *bom* sequence in pJH18 is critical to prevent plasmid loss during bacterial growth in tumor tissue.

To further assess the antitumor activity of ClyA expressing bacteria, C57BL/6 mice bearing B16F10 were treated with PBS, attenuated *S. typhimurium* harboring empty vector pJH18 (SAM-E) or pJH18-CR (SAM-CR) through intravenous injection, respectively. Mice revealed a decrease in body weight after bacterial treatment compared with PBS treatment, which might be due to inflammation after bacterial treatment (Fig. S3A). The best therapeutic effect was monitored in mice treated with SAM-CR, and the moderate suppression was observed in mice treated with SAM-E (Fig. S3B and C). These data demonstrated that engineered bacteria secreting ClyA triggered antitumor activity.

We finally evaluated whether *in vivo* gene expression in the SAM-CR strain could be enhanced by a lower dose of Doxy inducer. When induced with a 10-fold lower dose of Doxy (1.7 mg/kg body weight), bioluminescence was strongly detected in tumors colonized by SAM-CR but not in those colonized by SAM-pJL87 (10-fold higher in the former than in the latter) (Fig. S4A and B). This result indicates that gene expression could be enhanced by transforming bacteria with the novel Doxy-inducible pJH18 system.

## Discussion

This report describes our engineering of a novel Doxy-inducible gene expression system (pJH18) for bacteria-based cancer therapy in which multiple proteins are expressed in a balanced way by a single vector containing the bidirectional promoters P_*tetA*_ and P_*tetR*_. In particular, decoupling the *tetR* gene from P_*tetR*_ and placing it under the control of a weak constitutive promoter (P_*araB*_) resulted in promotion of transcriptional activity of target genes under P_*tetR*_. Moreover, the location of a *bom* sequence in *tet* expression system, which is required for plasmid mobilization, plays a critical role in preventing bacterial plasmid loss under normal conditions, ensuring maintenance of durable protein expression.

In this study, we demonstrate that the direct regulation of cargo by P_*tetR*_ and the constitutive expression of an appropriate amount of the repressor protein TetR play crucial roles in tightly regulating gene expression in response to Doxy and in maintaining a balance in the expression of target genes by P_*tetA*_ and P_*tetR*_. These results provide a proof-of-principle for a novel Doxy-inducible system (pJH18) to potentiate cargo expression in tumor-targeting *S. typhimurium*. In fact, the engineered Doxy-inducible pJH18 system established only around a 3-fold difference in promoter strength between P_*tetA*_ and P_*tetR*_ in comparison with a 100-fold difference with the pJL39 system. In previous systems (pJL39 and pJL87) [8], TetR proteins are controlled directly by P_*tetR*_ promoter bound to *tetO* operators to block RNA polymerase binding and subsequently inactivate gene transcription by P_*tetA*_ and P_*tetR*_ in the Doxy-free condition (Tet-OFF) [8, 28]. In the presence of Doxy, the P_*tetR*_ of pJL39 and pJL87 has to initiate constitutive production of TetR repressors, as well as downstream cargo genes, and therefore, a sufficient amount of Doxy (10 ng/ml) is required to trigger conformational changes in TetR to activate gene expression (Tet-ON) [8]. Furthermore, pJL39 system often caused to express a distal cargo gene such as *rluc8* gene much less than a proximal one such as *tetR* because its operonic structure consisted of a proximal *tetR* and a distal cargo gene driven by P_*tetR*_ promoter [8, 29, 30]. Thus, we modified pJL39 to generate pJH18, in which P_*tetA*_ and P_*tetR*_ are placed in close proximity to the 5′-end of genes of interest (GOIs). Such expression system is considered the desired consensus sequence for a higher-affinity RNA polymerase site, leading to improvement of transcriptional activity [8, 30].

As recent cancer therapies tend to address approaches using combination therapy [14], a multiplex expression system is desirable in anticancer bacterial engineering to provide diverse therapeutic mechanisms from a single programmed bacterial strain [8, 29, 31]. A single vector system for co-expression of multiple cargos can be achieved by using either multiple expression cassettes or a single expression cassette (polycistronic or monocistronic) [8]. With the multi-cassette approach, differences in the rate of transcription, translation, and stability of RNA and protein products can result in imbalances in the amounts of the protein products. With the single-cassette-polycistronic system, the distal genes/ORFs often show less efficient expression than the proximal genes, and there can also be considerable variation depending on the bacterial strain and the growth conditions [8, 32]. The present pJH18 system containing divergent promoters to directly regulate proximal genes should be beneficial for various applications requiring strong release of synergistic multiple genes in a balanced manner. Further study using bacteria that produce multiple anticancer cargos is underway, and the therapeutic effects and mechanisms of action are under evaluation.

A lower Doxy level might be better in gene expression induction, as it could reduce potential side effects, because Doxy is an antibiotic used in human patients with infectious disease [33]. In pJH18 system, low expression of TetR by constitutive weak promoter P_*araB*_ is able to block transcription through binding of TetR to *tetO* operator in the absence of Doxy. Therefore, much less Doxy (10~20 ng/ml for *in vitro* use) is required to trigger conformational change in TetR, dissociation from *tetO*, and eventual gene expression. The *in vitro* result was reproduced by an *in vivo* study in which bioluminescence of SAM-CR in CT26 xenografts was 10-fold stronger than that of SAM-pJL87 when induced with a 10-fold lower dose of Doxy (1.7 mg/kg body weight).

Bacteria as an anticancer drug delivery vehicle require durable plasmid inheritance to maintain gene expression through cellular generations. However, plasmid replication and gene expression utilizing the host machinery may result in plasmid extinction in the absence of a selective pressure such as that created by the presence of antibiotics [34]. Plasmid mobilization elements play a major role in the intracellular transfer of bacterial plasmids, and among them, *bom* sequence is known as a recognition site for the initiation of transfer [26, 35]. In fact, this sequence is necessary for plasmid stability during cell division, and it prolongs durable cargo expression in both *in vitro* and *in vivo* applications.

In conclusion, we successfully developed a method for doxycycline-induced co-expression of two proteins at similar expression levels, whereby we exploited bioluminescence reporter proteins with preclinical but no clinical utility. Future validation with clinically compatible reporter systems, for example, suitable for radionuclide imaging, is necessary to develop this system further towards potential clinical application. On the basis of this engineering, we have developed programmed bacterial strains that enable tight regulation of a wide variety of anticancer agents for advanced bacteria-mediated cancer therapy for a broad range of cancer patients.

## Materials and Methods

### Bacterial Strains and Cancer Cell Line

*E. coli* DH5a-competent cells were purchased from Enzynomics (Daejeon, Korea) and used for all gene cloning work. The SAM bacterium was a ΔppGpp-defective *S. typhimurium* SHJ2037 (*relA*::*cat*, *spoT*::*kan*) strain that is used to measure gene expression and activity in various plasmids [36]. All bacteria were grown in Luria-Bertani (LB) broth at 37 °C with agitation.

A murine colon carcinoma CT26 cell line was purchased from the American Type Culture Collection (MD, USA). The cells were cultured in high-glucose Dulbecco’s Modified Eagle Medium (DMEM) (Welgene, Gyeongsangbuk-do, Korea) containing 10 % fetal bovine serum (FBS) and 1 % penicillin–streptomycin and were incubated at 37 °C in an incubator with 5 % CO_2_.

### Plasmid Construction of a Divergent Gene Expression System Controlled by Doxy

pJH18, a plasmid with novel bidirectional *tet* promoter (P_*tetA*_ and P_*tetR*_), was engineered as follows. Using pJL39 plasmid as a template [8, 37], the *tetR* OFR [37] was amplified by polymerase chain reaction (PCR) with a TetR-R(EcoRI) and TetR-F(XbaI) primer set. After digestion with *Eco*RI and *Xba*I, the fragment was introduced into the same enzyme sites of pBAD plasmid and named pBAD-TetR [9]. The plasmid has *tetR* ORF driven by P_*BAD*_ promoter. The fragment containing a bidirectional *tet* promoter and multiple cloning sites at both ends (MCSI and MCSII) was amplified by PCR using a Tet-F(AflII) and Tet-R(HindIII) primer set with pJL39 as a template. The amplified fragment was digested with *Afl*II and *Hin*dIII and inserted into the same enzyme sites of pBAD-TetR, which was called pTet-BAD. The *bom* fragment was amplified by the bom-F(PciI) and bom-R(NheI) primer set using pBAD-Rluc8 as a template and was cloned between *Pci*I and *Nhe*I enzyme sites of pTet-BAD after enzyme digestion. It was named pTetII which has lost P_*BAD*_ promoter. Finally, a constitutive promoter P_*araB*_ was amplified by a P_*araB*_-F(MfeI) and P_*araB*_-R(MfeI) primer set using *E. coli* DH5α genomic DNA as a template and was cloned upstream of *tetR* ORF in pTetII using the Gibson assembly method (New England Biolabs, MA, USA). The resulting plasmid was named pJH18 and used it as a backbone in this study.

Various cargo gene fragments were amplified by PCR. The *rluc8* gene was driven by P_*tetA*_ promoter (P_*tetA*_::*rluc8*) and was amplified by an Rluc8/ClyA-P_*tetA*_-F(StuI) and Rluc8-P_*tetA*_-R(SpeI) primer set using the pBAD-Rluc8 plasmid as a template. The *rluc8* gene driven by P_*tetR*_ promoter (P_*tetR*_::*rluc8*) was amplified by an Rluc8-P_*tetR*_-F(KpnI) and Rluc8-P_*tetR*_-R(HindIII) primer set using the pBAD-Rluc8 plasmid as a template.

The *clyA* gene driven by P_*tetA*_ promoter (P_*tetA*_::*clyA*) was amplified by an Rluc8/ClyA-P_*tetA*_-F(StuI) and ClyA-P_*tetA*_-R(SacI) primer set using the pBAD-ClyA plasmid as a template. The *clyA* driven by P_*tetR*_ promoter (P_*tetR*_::*clyA*) was amplified by a ClyA-P_*tetR*_-F(KpnI) and ClyA- P_*tetR*_-R (SalI) primer set using the pBAD-ClyA plasmid as a template.

The PCR fragments were digested by the indicated restriction enzymes and cloned into the corresponding sites in pJH18. The resulting plasmids were named pJH18-RR (P_*tetR*_::*rluc8*), pJH18-AR (P_*tetA*_::*rluc8*), pJH18-CR (P_*tetA*_::*clyA,* P_*tetR*_::*rluc8*), and pJH18-RC (P_*tetA*_::*rluc8,* P_*tetA*_::*clyA*).

To evaluate the expression level of distal genes/ORFs, we developed pBAD-ClyA-Rluc8 from pBAD-ClyA system. The *rluc8* gene was amplified by an Rluc8-P_*BAD*_-F(SalI) and Rluc8-P_*BAD*_-F(SphI) primer set using the pBAD-Rluc8 plasmid as template. The PCR products were digested by the indicated restriction enzymes and cloned into the corresponding sites in pBAD-ClyA for generating pBAD-ClyA-Rluc8 (P_*BAD::clyA::cluc8*_).

The primers and plasmids used in cloning were shown in Tables S1 and S2 and Fig. 1.

### Western Blot Analysis and Luciferase Activity Assay

For western blot analysis, SAM bacteria transformed with the plasmids were incubated overnight in LB medium containing ampicillin (100 μg/ml). The cultured bacteria were inoculated into a medium with 1:100 dilutions and cultured. At an optical density of 0.6 at 600 nm (OD_600_), Doxy [0–500 ng/ml in ethanol (w/v)] was added, and the bacteria were further cultured. After 4 h, the bacteria were precipitated with centrifugation at 4000 rpm for 5 min. After simple washing with phosphate-buffered saline (PBS), the bacterial pellets were dissolved in sodium dodecyl sulfate (SDS) buffer containing 0.2 % β-mercaptoethanol (Sigma-Aldrich, Darmstadt, Germany) and were heat-treated for 5 min at 95 °C. The dissolved bacterial proteins (0.5 OD_600_ equivalents per lane) were separated in 15 % SDS-PAGE (40 % Acrylamide, Tris-HCl (pH 6.8)/Tris base (pH 8.8), SDS, ammonium persulfate (APS), TEDMED) in running buffer (Tris base, glycine, sodium dodecyl sulfate) and then transferred to nitrocellulose membrane (GE Healthcare, MA, USA) by transfer buffer (Tris base, glycine). The membranes were incubated in blocking buffer [5 % (w/v) skim milk in Tris-buffered saline buffer (Tris base, NaCl) containing 0.1 % Tween 20 (Sigma-Aldrich, Darmstadt, Germany) (TBS-T)] at room temperature for 1 h. After decanting the blocking buffer (TBS + 5 % w/v skim milk), primary antibodies in 1X TBS with 1 % skim milk were added to the membranes, and they were incubated for 2 h. After decanting the primary antibodies and simple washing with TBS-T (500 ml TBS 1X + 500 μl Tween) (three times), secondary antibodies conjugated with horseradish peroxidase (HRP) were added to the membranes and incubated for 1 h. Before adding the chemiluminescence HRP substrate (Merck Millipore, MA, USA), the membranes were washed with TBS-T three times. The specific protein bands against each antibody were detected with a ChemiDoc^TM^ XRS+ system imager (BIO-RAD, California, USA). All antibodies used in this study are listed in Table S3.

For the luciferase activity assay, SAM bacteria transformed with the pJH18 plasmids containing the *Rluc8* gene were induced by increasing the concentration of Doxy in the early exponential phase (OD_600_, 0.5~0.7) for 4 h. The bacterial pellets (OD_600_, 0.5 per sample) were resuspended in 100 μl of PBS to measure Rluc8 activity immediately after addition of 5 μl of coelenterazine (0.2 μg/μl) at room temperature. Light emission was measured using a Microlumat Plus LB96V luminometer (Berthold Technologies, Bad Wildbad, Germany) or a Lumina S5 *in vivo* imaging system (IVIS; Perkin Elmer, MA, USA) with an exposure every 1 s. The measured values were calculated as relative luminescence units [38].

### Measurement of Plasmid Loss During Bacterial Culture

SAM bacteria transformed with the indicated plasmids (pBAD-clyA, pJH18-CR (without *bom* sequence), pJL87, pJH18-CR) were first grown overnight on LB agar plates containing ampicillin. Single colonies from plates were inoculated into 5-ml LB broth without ampicillin at 37 °C with shaking. After 1 day, the bacteria were serially 10-fold diluted with LB broth, and 100 μl of the diluted bacteria were spread out on a plate with or without ampicillin and incubated overnight at 37 °C. The numbers of colonies in each plate were counted the next day.

### Animal Grouping and Housing, Mouse Tumor Model, and Bacterial Injection

Female 6-week-old BALB/c mice and C57BL/6 (~20 g in body weight) were purchased from Orient (Seongnam, Korea) and keep in specific pathogen-free conditions of mouse room for 1 week prior to starting any experiments. The mice were housed in a group of 3–5 mice in per plexiglass cage (22.3 × 26.8 × 13.0 cm) under 12h/12h at light/dark cycle (light on at 7:00 am) in a temperature-controlled room (22– 24 °C) and 40–60 % humidity, with free access to tap water and standard chow diet (PMI Nutrition International, LLC 4001 Lexington Avenue North, Arden Hills, MN 55126). Cages were lined with full autoclave wood fiber (Northeastern Product Corp., Warrensburg, NY, 12885) and replaced by clean cages 2 times/week. No enrichment material was used inside the cage. At the moment of the experiments, mice were weighing approximately 18 g and aging 7 weeks. The animals were randomly selected from different cages to house them in one new cage for tumor implantation, and each mouse was used for experiment only once. All experimental processes were performed from 11:00 to 7:00 pm. After culture in DMEM media, CT26 cells (10^6^ cells per mouse) or B16F10 cells (5 × 10^5^ cells per mouse) were detached and subcutaneously injected into mice anesthetized with 2 % isoflurane. After cell injection, it took about 12 days for the tumor volume to reach 150–180 mm^3^. All animal care and experimental procedures were performed following the guidelines of the Animal Care and Use Committee, Chonnam National University (Gwangju, Korea), the National Centre of the Replacement, Refinement and Reduction on Animals in Research [39].

For bacterial injections, SAM transformants cultured overnight were inoculated into fresh LB media containing ampicillin in a 100-fold dilution ratio and were further incubated until an OD_600_ of 2~2.5 (early stationary phase) was attained. After centrifugation at 4000 rpm for 5 min, bacterial pellets were washed with PBS twice. Bacteria (3 × 10^7^ CFU/100 μl in PBS) were intravenously injected into the mice *via* the tail vein. To induce cargo genes in SAM transformants, the indicated amounts of Doxy (Sigma-Aldrich, MO, USA) were orally administered using gavage (starting at 3-day postinoculum, once a day after 1 h of fasting) [8]. Tumor volume (mm^3^) was calculated using the formula (length × height × width)/2 of the tumor in millimeters [8]. Most data analysis and experiments were blinded to prevent any bias.

### *In Vivo* Bioluminescence Imaging and Detection of the Cargo Molecule in Tumor Tissues

To obtain bioluminescence images 12 h after oral Doxy administration, tumor-bearing mice were anesthetized with 2 % isoflurane and then received coelenterazine (0.7 mg/kg body weight) through intravenous injection. After 1 min, bioluminescence imaging was acquired from the mice using an IVIS system, as described previously [17, 40].

To measure the existence of cargo molecules, tumor tissues were obtained from mice and homogenized in protein extraction solution (20 mM Tris-Cl pH 6.8, 150 mM NaCl, 5 mM EDTA, 1 % NP-40, protease inhibitor 1X (Xpert protease inhibitor cocktail solution 100X, GenDEPOT)). The homogenized tissue samples were centrifuged and filtered through a 0.2-μm filter. A total of 20 μg of proteins were separated using 15 % SDS-PAGE, and western blot analysis was performed with specific antibodies.

### Bacterial Counting in Tumor Tissues

After sacrificing the mice, tumors and other organs were imaged with IVIS. Tumors were homogenized and filtered as the sample preparation for the western blot analysis. The filtrates were serially diluted with PBS and spread out on LB agar plates with or without ampicillin. After overnight culture, the bacteria were enumerated, and the results were expressed as CFU/g of organ.

### Statistical Analysis

Statistical analysis was performed using GraphPad Prism 8.0 software with *p* < 0.05 considered significant. Two independent groups were compared using unpaired two-tailed *t*-tests. Comparison of multiple experimental groups was evaluated using one- or two-way ANOVA with multiple comparisons *post hoc* test to obtain multiplicity-adjusted *P* value. All data are shown as mean ± SEM.

## Supplementary Information

Fig. S1. Comparison of cargo expression in SAM-CR, SAM-RC and SAM transformed with pBAD-Rluc8 or pBAD-ClyA. (JPG 263 kb)

Fig. S2. Comparison of cargo co-expression in SAM-CR and SAM transformed with pBAD-ClyA-Rluc8 (SAM-pBAD-CR). (JPG 213 kb)

Fig. S3. Efficacious antitumor responses of bacteria secreting ClyA in B16F10 melanoma tumor model. (JPG 346 kb)

Fig. S4. Expression efficiency of pJH18 transformed bacteria after low dose Doxy administration in tumor-bearing mice. (JPG 247 kb)

Table S1. List of primers used in the study. (JPG 263 kb) (JPG 289 kb)

Table S2. Bacterial strains and plasmid used in the study (JPG 135 kb)

Table S3. Antibodies used in the study. (JPG 153 kb)
