# Supplementary figures and images for: Optimized Doxycycline-Inducible Gene Expression System for Genetic Programming of Tumor-Targeting Bacteria
Source: Mol Imaging Biol. 2021 Aug 17;24(1):82–92. doi: 10.1007/s11307-021-01624-x (PMC8760206; doi:10.1007/s11307-021-01624-x)

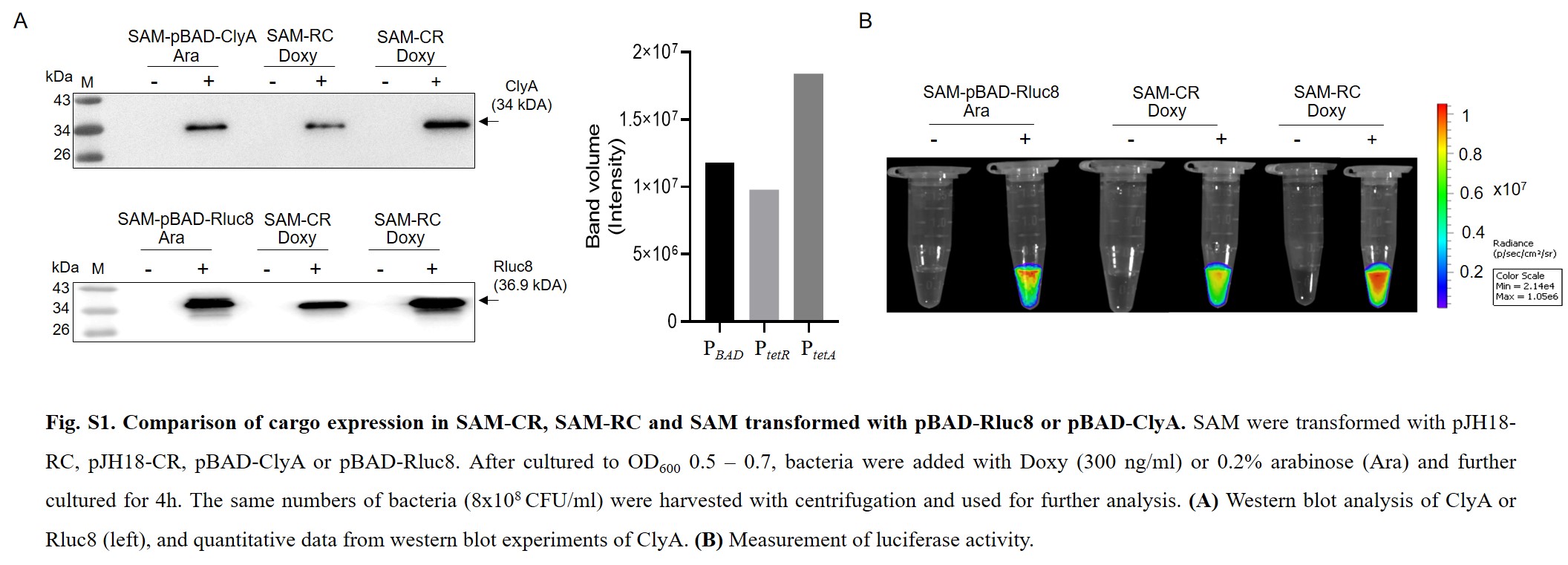

Supplement: Supplementary file 1 — Comparison of cargo expression in SAM-CR, SAM-RC and SAM transformed with pBAD-Rluc8 or pBAD-ClyA. (JPG 263 kb) [file 11307_2021_1624_MOESM1_ESM.jpg]

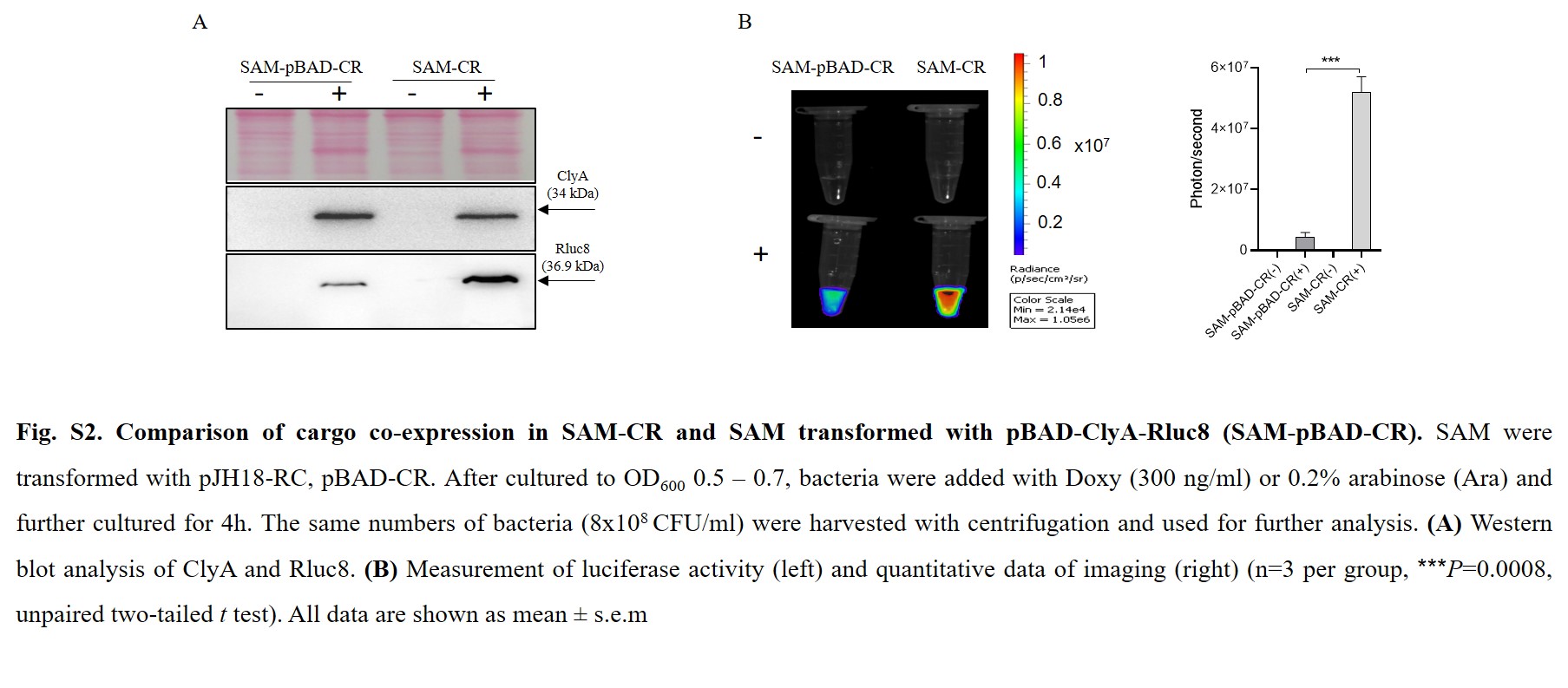

Supplement: Supplementary file 2 — Comparison of cargo co-expression in SAM-CR and SAM transformed with pBAD-ClyA-Rluc8 (SAM-pBAD-CR). (JPG 213 kb) [file 11307_2021_1624_MOESM2_ESM.jpg]

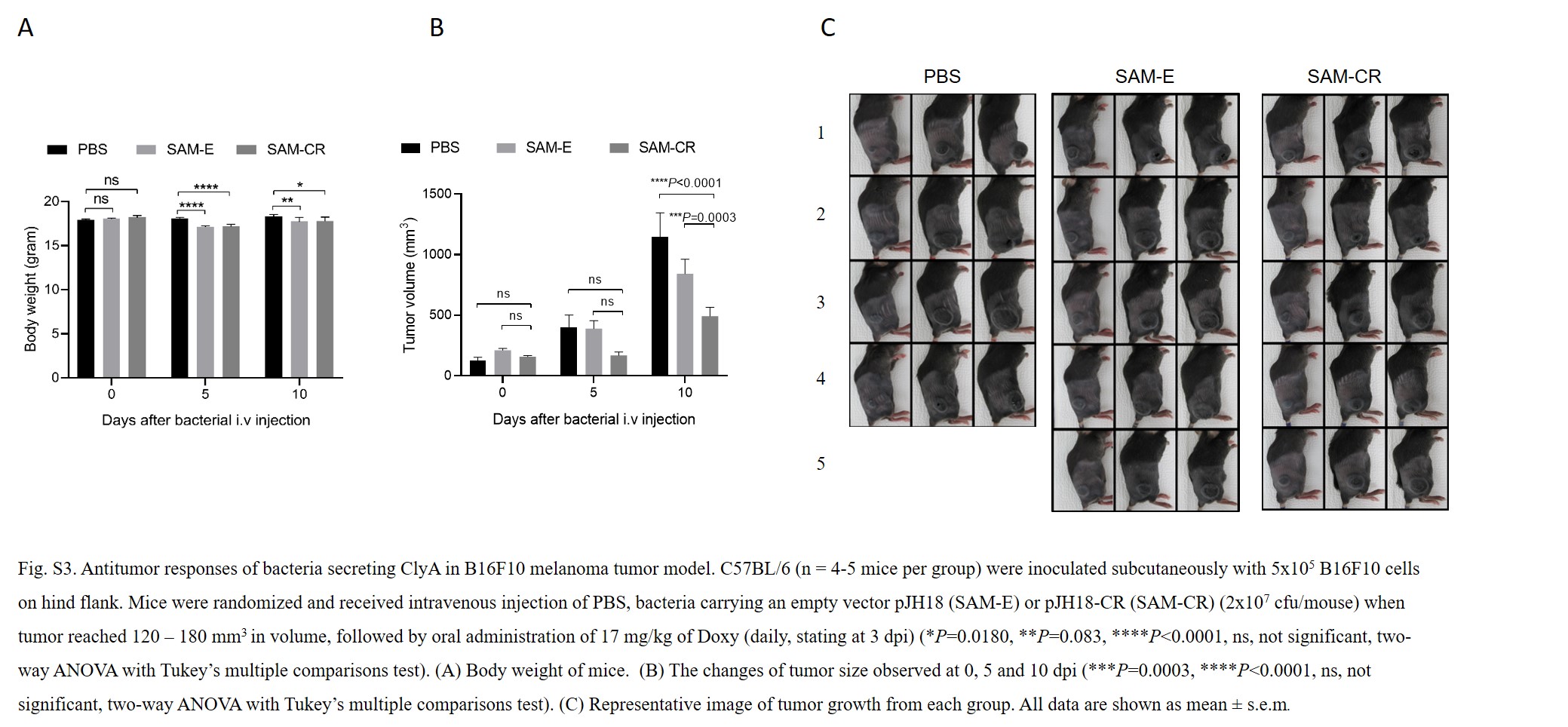

Supplement: Supplementary file 3 — Efficacious antitumor responses of bacteria secreting ClyA in B16F10 melanoma tumor model. (JPG 346 kb) [file 11307_2021_1624_MOESM3_ESM.jpg]

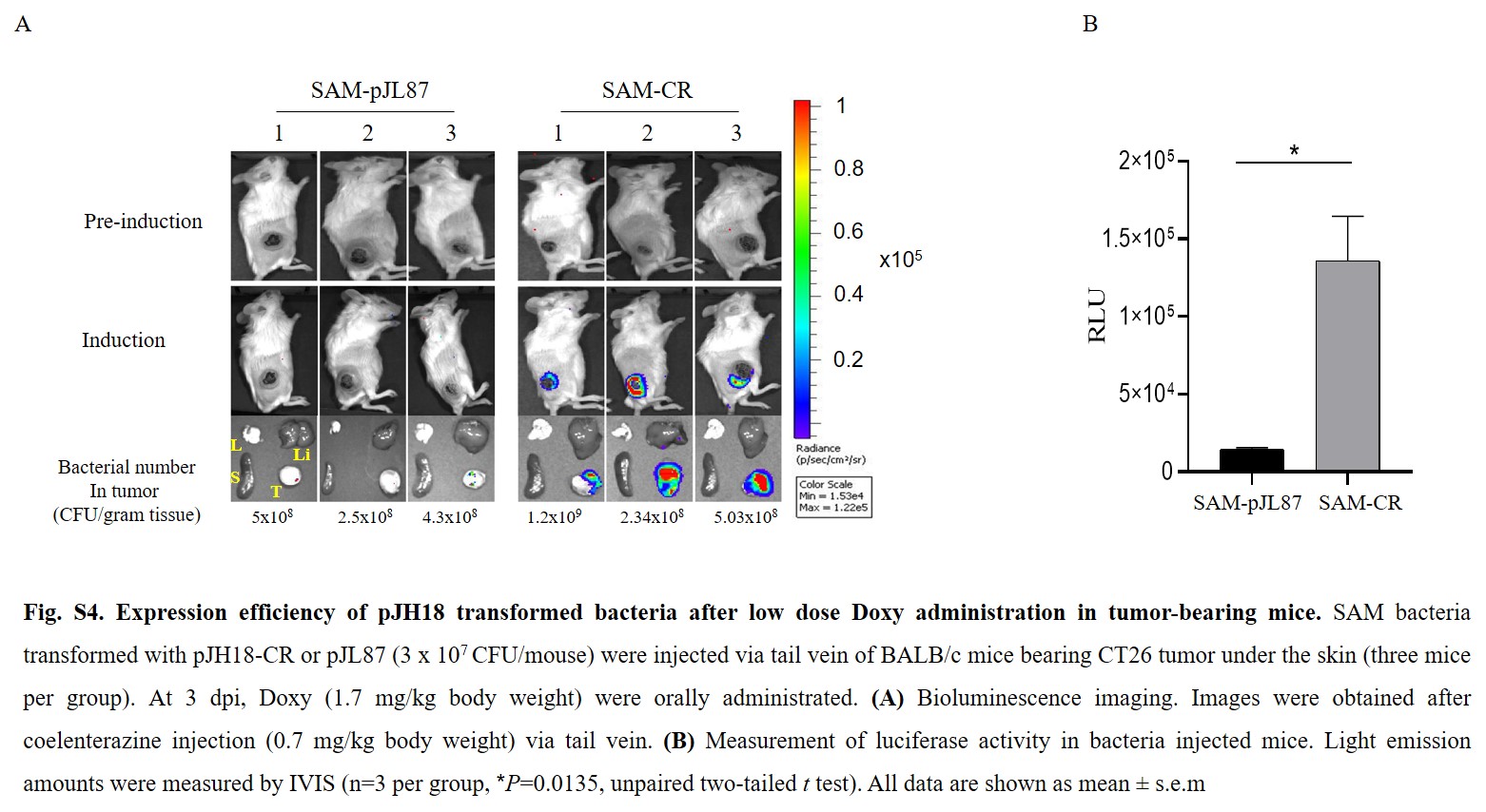

Supplement: Supplementary file 4 — Expression efficiency of pJH18 transformed bacteria after low dose Doxy administration in tumor-bearing mice. (JPG 247 kb) [file 11307_2021_1624_MOESM4_ESM.jpg]

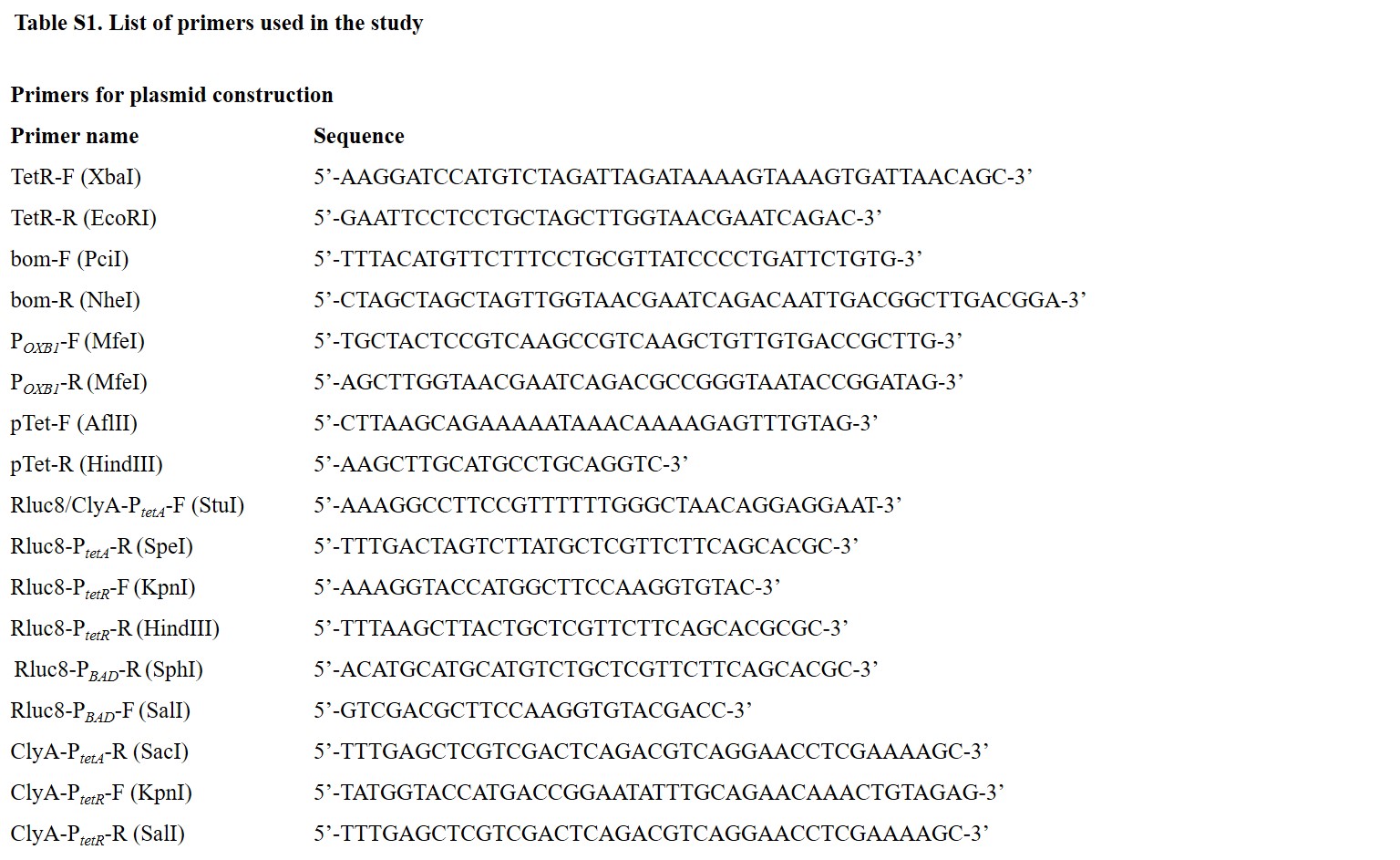

Supplement: Supplementary file 5 — List of primers used in the study. (JPG 263 kb) (JPG 289 kb) [file 11307_2021_1624_MOESM5_ESM.jpg]

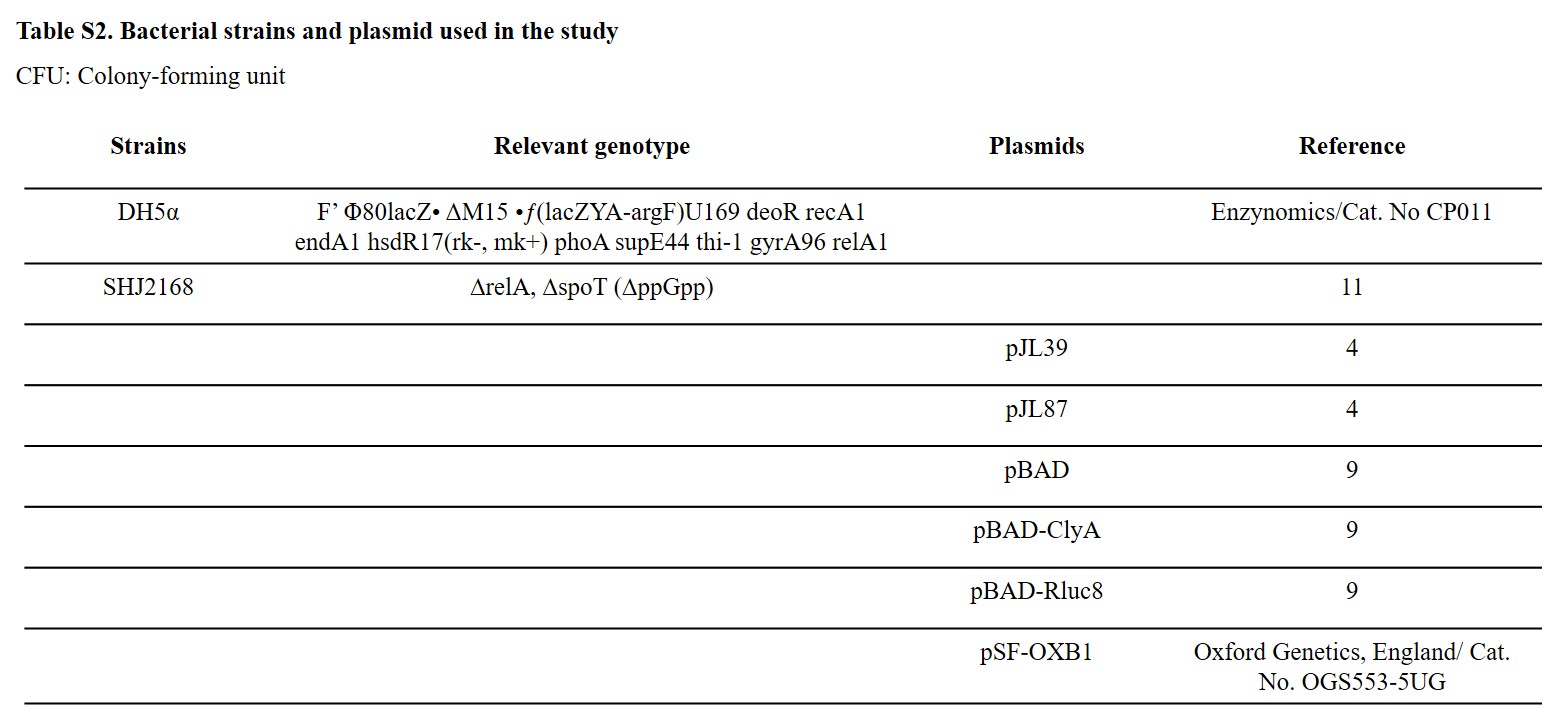

Supplement: Supplementary file 6 — Bacterial strains and plasmid used in the study (JPG 135 kb) [file 11307_2021_1624_MOESM6_ESM.jpg]

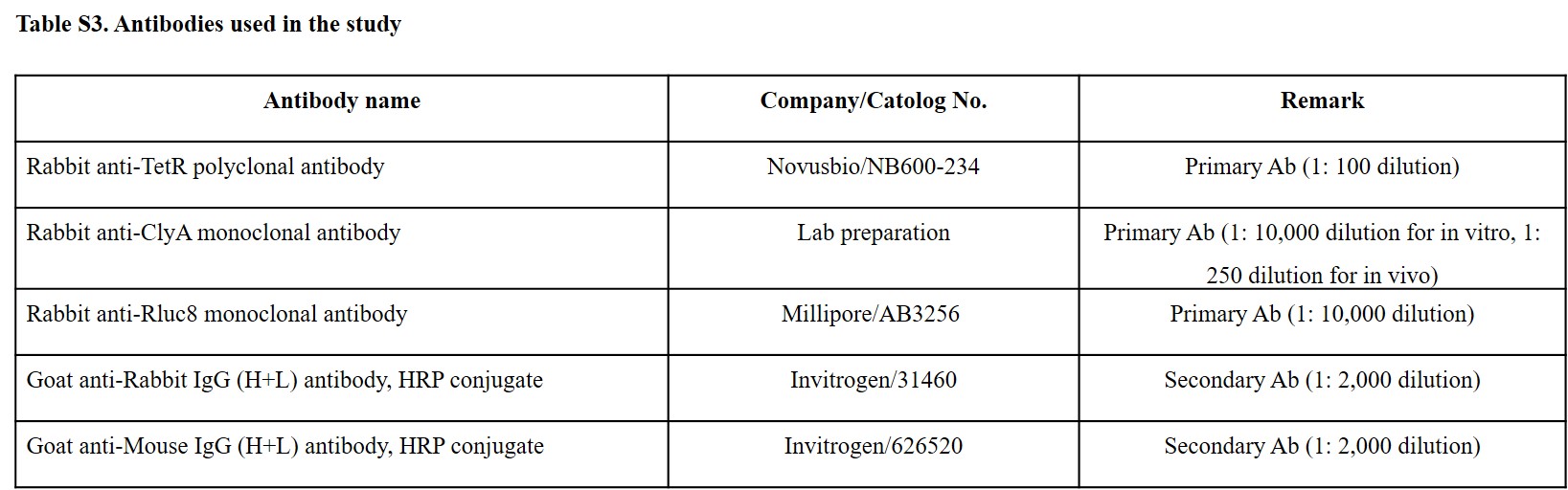

Supplement: Supplementary file 7 — Antibodies used in the study. (JPG 153 kb) [file 11307_2021_1624_MOESM7_ESM.jpg]
